# Analysis of Cytochrome P450 Conserved Sequence Motifs between Helices E and H: Prediction of Critical Motifs and Residues in Enzyme Functions

**DOI:** 10.4172/2157-7609.1000110

**Published:** 2011-08-30

**Authors:** Numan Oezguen, Santosh Kumar

**Affiliations:** 1 Internal Medicine-Endocrinology, University of Texas Medical Branch, 301 University Blvd., Galveston, TX 77555-1060, USA; 2 Division of Pharmacology and Toxicology, School of Pharmacy, University of Missouri-Kansas City, 2464 Charlotte St., Kansas City, MO, USA

**Keywords:** Cytochrome P450, Conserved sequence motif, Directed evolution, Site-directed mutagenesis, Structure-function relationships

## Abstract

Rational approaches have been extensively used to investigate the role of active site residues in cytochrome P450 (CYP) functions. However, recent studies using random mutagenesis suggest an important role for non-active site residues in CYP functions. Meta-analysis of the random mutants showed that 75% of the functionally important non-active site residues are present in 20% of the entire protein between helices E and H (E-H) and conserved sequence motif (CSM) between 7 and 11. The CSM approach was developed recently to investigate the functional role of non-active site residues in CYP2B4. Furthermore, we identified and analyzed the CSM in multiple CYP families and subfamilies in the E-H region. Results from CSM analysis showed that CSM 7, 8, 10, and 11 are conserved in CYP1, CYP2, and CYP3 families, while CSM 9 is conserved only in CYP2 family. Analysis of different CYP2 subfamilies showed that CYP2B and CYP2C have similar characteristics in the CSM, while the characteristics of CYP2A and CYP2D subfamilies are different. Finally, we analyzed CSM 7, 8, 10, and 11, which are common in all the CYP families/subfamilies analyzed, in fifteen important drug-metabolizing CYPs. The results showed that while CSM 8 is most conserved among these CYPs, CSM 7, 9, and 10 have significant variations. We suggest that CSM8 has a common role in all the CYPs that have been analyzed, while CSM 7, 10, and 11 may have relatively specific role within the subfamily. We further suggest that these CSM play important role in opening and closing of the substrate access/egress channel by modulating the flexible/plastic region of the protein. Thus, site-directed mutagenesis of these CSM can be used to study structure-function and dynamic/plasticity-function relationships and to design CYP biocatalysts.

## Introduction

Understanding the molecular basis of diverse functions of mammalian cytochrome P450s (CYPs), which will enable to predict drug metabolism and drug interactions, is critical [1–5]. However, despite sequence variability (20% – 99%), the similarity in tertiary structure of CYPs makes it difficult to examine their diverse functional characteristics [6–10]. From the mid1990s to early 2000 rational approaches, such as chimeragenesis, homology modeling, and site-directed mutagenesis have been extensively used to examine CYP structure-function relationships [8–11]. However, these studies targeted only substrate recognition site (SRS) or active-site residues.

Crystal structures of several CYP enzymes exhibit structural diversity of active as well as some non-active site regions, both of which may be responsible for functional diversity [12–17]. Furthermore, findings using X-ray crystallography, isothermal titration calorimetry (ITC), and NMR have revealed that CYPs access and bind substrates/inhibitors of different size/shape through ligand-induced conformational change, suggesting the role of non-active site regions [14,18–19]. These findings are consistent with reports, including ours using directed evolution approach, that non-active site residues also play a critical role in substrate specificity and selectivity, in addition to enzyme activity and stability [20,21]. Since understanding of the functional role of individual non-active site residues is difficult using only rational or directed evolution approaches, there is a critical need for alternate approaches to identify the role of non-active site residues in enzyme activity and stability, as well as substrate/inhibitor selectivity.

Recently, we utilized an alternate approach, analysis of conserved sequence motifs (CSM), which resulted in the identification of twenty CSM in the CYP2 family [22]. We investigated CSM 8 (E-F loop) because it is present between plastic/variable regions 3 (177–188) and 4 (203–298) (14) and may regulate ligand-induced flexibility. Mutation of CSM 8 residues Arg^187^, Phe^188^, Tyr^190^, and Asp^192^, which have the highest degree of residues conservation, to Ala revealed a preference for larger ligands over smaller ligands. These mutants also increased the dynamics of the protein leading to decreased thermal stability. Therefore, in the current study we extended our CSM analysis to CYP families 1, 2, and 3, as well as CYP2 subfamilies 2A, 2B, 2C, 2D, and 2E, which contain important drug- and xenobiotic-metabolizing CYP enzymes. Based on analysis of each CSM between helices E and H (E-H) and residues within the CSM, we predicted critical CSM residues that may be involved in enzyme functions, or alter the opening/closing dynamics and overall stability that affects enzyme function.

## Methods

### Identification of conserved sequence motifs

We used a similar approach to that described in our recent work for the CYP2 family [22]. Briefly, we selected 19 CYP1 and 26 CYP3 sequences from species, such as human, mouse, and rat, and generated a multiple sequence alignment with ClustalW [23]. Similarly, 12 CYP2A, 10 CYP2B, 33 CYP2C, and 25 CYP2D sequences were utilized from various species, as presented earlier [22], and generated a multiple sequence alignment with Clusta1W. These multiple sequence alignments were further analyzed using PCPMer, in which each of the 20 natural amino acids was represented by a 5-dimensional vector [24]. The vectors of 5-dimensional space were derived by 237 dimensional physicochemical property (PCP) space. The eigenvector that had the highest eigenvalue (r=0.95) was correlated with the hydrophilicity scale. Furthermore, PCPMer generated a profile for the alignment at every position, which included the standard deviation and relative entropy for each position and component of the 5-dimensional space. PCPMer then used these profiles to identify high relative entropy clusters (highly conserved regions) and were indicated as rank. Finally, three different levels of conservation within the motifs were manually as well as computationally identified based on the presence of identical residues. They were defined as highly conserved (>90%), intermediate conserved (75–90%), and least conserved (<75%) residues.

## Results and Discussion

### Analysis of the functional residues of CYPs identified by random mutagenesis

We analyzed CYP mutants that were obtained by a random mutagenesis/directed evolution approach with CYP1A2, CYP2A6, CYP2Bs, and CYP3A4 (Table 1) [25–38]. The results clearly showed that approximately 85% of the beneficial mutants are in the regions between helices E and I (E–I, ~125 amino acids), which occupy only 25% of the entire protein. However, the remaining proteins having mutations, which include D helix, K′-L loop, J-J′ loop, and L helix, contain only 15% of the functionally important residues. Another observation from this analysis was that except for residues 209 and 305 of CYP2A6, the other seven residues belong to non-active sites. Furthermore, directed evolution identified four CYP2A6 residues (287, 297, 300, and 305) and one CYP2B1 residue (295) in the I-helix that have been described as the backbone of the protein and that belong to substrate recognition site 4 (SRS4). Therefore, many SRS 4or I- helix residues in CYP2Bs (289, 290, 292, 294, 297, 298, 302) have been extensively studied using rational approaches for structure-function relationships [9,39–44]. Similarly, the F-G region (SRS 2), which is considered part of the substrate access channel in P450 51 enzyme, has been studied using rational approaches in CYP2B1. However, the minimal changes in biochemical characteristics of CYP2B1 F-G mutants do not support access via the F-G region of CYP2B1, and suggest the alternate access route identified in P450 51. Therefore, further study in CYP2B1 helix B′ flexible region using rational mutagenesis suggests that residues in the helix B′ region affect regio- and stereoselective oxidation in CYP2B enzymes as well as substrate entry [45]. Furthermore, molecular modeling and substrate docking studies in CYP2B enzymes clearly suggest the role of B′ helix/B′-C loop in substrate access/egress channel [46]. The SRS 2 residues, 206 and 209, in CYP2B1 have been shown critical for CYP expression and activity [40,42,47]. These residues have also been identified as functionally important in CYP2A4 and CYP2A5 [48–50].

**Figure 1:**
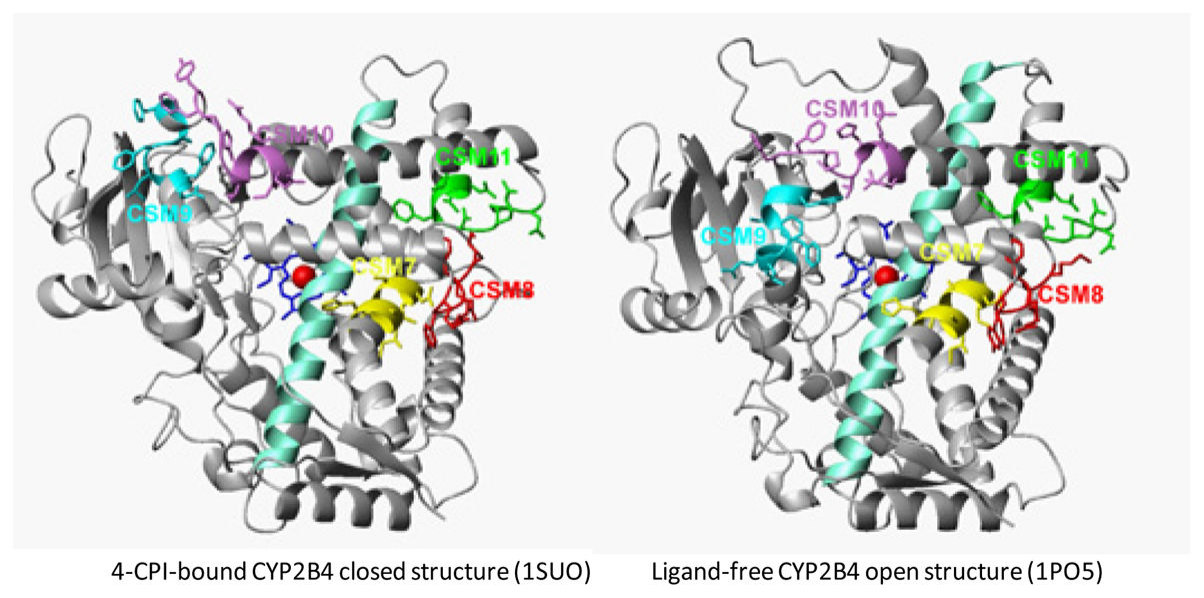
Open (ligand-free, 1PO5) and closed (4-CPI-bound, 1SUO) structures of CYP2B4 showing CSM 7–11 in the E-H region of the protein. The figures were generated using MOLMOL and Microsoft Publisher files. The CSM 7–11 are labeled and shown in different colors. The I-helix is shown in light green color.

Since I helix has been extensively studied using a rational approach and most of its functionally important residues have been identified, we further analyzed the regions between the helices E and H (E-H) using the CSM approach (Figure 1). The E-H region constitutes only 20% of the entire protein, but it contains 75% of the functionally important residues identified by random mutagenesis/directed evolution (Table 1). The E-H region contains five of the twenty CSM (7–11) in CYP2 family (22). The E-H region also contains an important flexible region between helices F to G (F-G) in CYP2Bs, CYP2Cs, and CYP3A4 identified by X-ray crystallography, ITC, and NMR (12, 14, 16, 18–19, 51). Although the F-G mutants do not suggest it as substrate channel [47], X-ray crystallography and ITC studies clearly suggest that the F-G region is critical for ligand-induced conformation adaptation.

### The CSM analysis of CYP1, CYP2, and CYP3 families

Since CYPs from the families 1, 2, and 3 are responsible for the metabolism of the majority of marketed drugs and other xenobiotics [4], we identified and analyzed CSM 7–11 of CYP1 and CYP3 families (Figure 1, Table 2). CSM 7–11 of CYP2 family has been reproduced from our previous work [22] for comparison. These comparisons yielded the following results: 1) CSM 9 is not present in the CYP1 and CYP3 families, suggesting a specific role for CSM 9 in the CYP2 family; 2) CSM 7 and 8 show a relatively higher rank (≥1.8) than CSM 10 and 11(1.4) in the CYP1 family; 3) all four CSM show a high rank (≥1.8) in the CYP3 family, which is similar to the rank in CYP2 family. Further analysis showed that the CSM 7, 8, 10, and 11 are conserved within families 1, 2, and 3. However, their amino acid residues are less conserved between these families. As an exception, CSM 8 of CYP 2 and CYP 3 contain 4 of the 6 identical residues and 2 other similar residues, suggesting that CSM 8 has a common role in the CYP2 and CYP3 families. Another striking observation is that even though CSM of CYP2 was analyzed from a large number of sequences (175), there is high amino acid conservation in CSM 7–11 compared to CYP 1 and CYP 3, in which small number of sequences were available for CSM analysis.

### The CSM analysis of CYP2A, 2B, 2C, 2D, and 2J subfamilies

Recently we identified several CSM in the CYP2A, CYP2B, CYP2C, CYP2D, and CYP2J subfamilies [22]. Here we further identified and analyzed CSM 7–11 in important drug- and xenobiotic-metabolizing CYP2A, CYP2B, CYP2C, and CYP2D subfamilies (Figure 1, Table 3). It can be noted that the CSM identified here is slightly different from the previous one in a way that CSM 9 (in this analysis) was considered specific to CYP2A, CYP2B, and CYP2D subfamilies (in the previous analysis) [22]. Overall, the results showed that while all the CSM (7–11) were present in CYP2B and CYP2C subfamilies, CYP2A contained CSM 8, 9, and 11 and CYP2D contained CSM 7, 8, and 9. On the other hand CYP2A contained two CSM that were specific to CYP2A (^204^MMLGIFQF^211^ and ^243^GLENF^247^). Overall, CSM 8 and 10 were common to all the CYP2 subfamilies and CSM 9 was present only in the CYP2B subfamily.

CSM 7, 8, and 9 showed a relatively higher ranks (>2.0) than CSM 10 and 11 (≤2.0) in both the CYP2B and CYP2C subfamilies. However, the rank of CSM in CYP2A and CYP2D were very low (≤ 1.2). CSM 8 showed 100% amino acid conservation, while CSM 10 had five of the six conserved residues when compared between CYP2B and CYP2C subfamilies. Overall, the levels of amino acid conservation between subfamilies CYP2B and CYP2C were in the order: CSM 8 > 10 > 7> 11 > 9 (Table 3). In contrast, all the CSM of CYP2A and CYP2D showed very poor amino acid conservation with the CYP2B and CYP2C subfamilies. These correlations suggest that the CYP2B and CYP2C enzymes have overlapping structural and functional characteristics, while CYP2A and CYP2D have unique structural and functional characteristics. Indeed, these results are strongly correlated with the fact that the CYP2B and CYP2C enzymes show remarkable flexibility in the E-H regions and can accommodate ligands of variable size and shape through ligand-induced conformational adaptation [12,14,16,18–19,51]. In contrast, CYP2A and CYP2D show small structural changes in these regions and can accommodate a small number of ligands with similar shape and size [52–56].

Strong conservation of the residues in CSM 8 and 10 between CYP2B and CYP2C strongly suggest that these residues have common functions in these subfamilies, perhaps in regulating the flexible regions of the protein and/or stabilizing the protein. In contrast, although CSM 7, 9, and 11 have a high rank, the residues in these CSM significantly differ between CYP2B and CYP2C subfamilies (Table 3). These observations suggest that these CSM have important and specific functions within the CYP2 subfamily, such as metabolism of specific substrates and enzyme cooperativity. This is consistent with the fact that CYP2C9 metabolizes small substrates and shows enzyme cooperativity, while CYP2C8 metabolizes large substrates and shows Michaelis-Menten kinetics [57–59]. Similarly, CYP2B enzymes generally do not show enzyme cooperativity and metabolize substrates of diverse size and shape [9,14].

Since CSM 8 is in the E-F loop and CSM 10 is in the G′-G loop in CYP2B4 structure, we propose that they act as a switch in regulating the flexibility of CSM 9 (F helix) and CSM 11 (H helix), respectively, in addition to the regions between these CSM. CSM 8 may also regulate the flexibility of CSM 7. This is consistent with the fact that CSM 7 is located at plastic region 3 (177–188) and CSM 9–11 are located at plastic region 4 (203–298) [14]. The proposed model is consistent with our recent studies with CYP2B4 CSM 8 mutagenesis, in which, we suggested the role of CSM 8 as a switch to regulate the flexibility of the F-G regions [22]. Further site-directed mutagenesis of CSM 7–11 followed by X-ray crytallography and ITC of the selected mutants can be performed with representative enzymes of the CYP2B (2B6) and CYP2C (2C9) subfamilies to identify the role of these CSM in regulating substrate/inhibitor selectivity, regio- and stereoselectivity, enzyme cooperativity, and protein stability. Similarly, to identify the role of CSM 7, 9, and 11 in substrate selectivity and enzyme cooperativity in CYP2B and CYP2C subfamilies, residues of CYP2B6 from these CSM can be swapped with the CYP2C9 residues followed by biochemical and biophysical characterizations.

### Analysis of CSM 7, 8, 10, and 11 in important drug- and xenobiotic-metabolizing enzymes of CYP1, 2, and 3 families

We analyzed CSM 7, 8, 10, and 11 in fifteen important human drug- and xenobiotic-metabolizing CYP enzymes (Table 4). The results showed that amino acid residues in CSM 8 are extremely conserved within CYP subfamilies (CYP 2A, 2B, 2C, 2D, and 2E) and are well conserved between the subfamilies. However, they are relatively less conserved between CYP1, CYP2, and CYP3 families. Similarly, amino acid residues in CSM 10 are highly conserved within CYP subfamilies, but relatively less conserved between the subfamilies. On the other hand, amino acid residues in CSM 7 and 11 are relatively less conserved compared to CSM 8 and 10 within and between the subfamilies/families. An intriguing finding is that the individual residues as well as number of residues in CYP1A enzymes are significantly different from the residues of CYP2A, CYP2B, CYP2C, CYP2D, CYP2E, and CYP3A enzymes at all the CSM. This can be explained from the fact that CYP2B, CYP2C, CYP2E, and CYP3A enzymes show flexibility and accept an array of ligands with different size, shape, and hydrophobicity [12,52,13–14,57,58,60]. On the other hand, CYP1A enzymes show the least flexibility in the E-H regions and they have compact active site structures as shown by X-ray crystal structure and homology modeling [54,55,61].

Although there are similarities in amino acid conservation among the CYP families and subfamilies, there are many differences in terms of amino acid identity. These similarities and differences may be associated with similar, overlapping, as well as unique characteristics of each CYP enzymes. For example, the third residue of CSM 8 is acidic (Asp or Glu) in most enzymes, however, CYP1A1 and CYP3A7 have Gln, CYP3A5 has Gly, and CYP2A6 and CYP2B6 have His. Similarly, the first residues of CSM 8 in most enzymes are Arg, but CYP1A1, CYP1A13, and CYP2E1 have His and CYP3A5 has Lys. Another intriguing observation is that the third residue of CSM 11 is Asp in all the enzymes, except CYP1A2 and CYP3As enzymes. While comparing all the CSM in CYP2C enzymes, we find that the residues in CSM7 are identical in all the CYP2C enzymes, while there are small differences in other CSM. For examples: 1) CYP2C19 has Phe^189^ instead of Tyr in CSM 8; 2) CYP2C8 has Cys^225^ instead of Tyr, CYP2C18 has Leu^226^ instead of Phe, and Ser^229^ instead of Thr in CSM 10;3) CYP2C9 has Gln^261^ instead of Arg and CYP2C18 has Ala^260^ instead of Pro in CSM 11. Similarly, while comparing residues at all the CSM in CYP3A enzymes, we find the following: 1) CYP3A7 has Ser^192^ instead of Arg in CSM 8; 2) CYP3A7 has Lys^224^ instead of Ile in CSM 9; 3) CYP3A5 has Leu^225^ instead of Val in CSM 10; 4) CYP3A5 has Lys^258^ instead of Glu, CYP3A7 has Gly^259^ instead of Ser, CYP3A7 has Glu^263^ instead of Asp, and CYP3A5 has Lys^264^ instead of Tyr in CSM 11. In addition, there are many differences in amino acid residues among CYP3A4, 3A5, and 3A7 at CSM 8, which is unusual compared with the CSM of other CYP families or subfamilies. These differences in amino acids either within or across the families/subfamilies may define unique role(s) for these CYP enzymes. The basis for these differences in the drug-metabolizing enzymes can be explored by swapping the amino acid residues in CYP enzymes between the families (e.g. CYP 2B6 vs. CYP3A4) and subfamilies (e.g. CYP2B6 vs. CYP2C9), as well as within the subfamilies (e.g. CYP2C8 vs. CYP2C9) and characterizing them for enzyme activity, substrate/inhibitor selectivity, substrate regio- and stereoselectivity, as well as enzyme cooperativity and stability.

In conclusion, the combination of directed evolution, X-ray crystal structures, and CSM analysis has provided evidence that the E-H regions are the most important regions in CYP structure-function and dynamic/plasticity–function relationships. More specifically, the CSM analysis of the CYP families, CYP2 subfamilies, and individual drug-metabolizing CYP enzymes suggested important structural and functional roles in terms of plasticity and dynamic aspects of CSM 7–11 in the E-H region, as well as important role of specific residues within these CSM. These CSM may play an important role in opening and closing of the substrate access/egress channel by modulating the flexible/plastic region of the protein and overall protein stability that affects enzyme function. The finding in the manuscript is significant and timely, because it is a step forward in understanding the complex nature of CYP structure-function relationships, especially in the flexible E-H regions, that are responsible for diverse drug metabolism and numerous drug interactions. Thus, further study using rational approaches of the E-H regions will help identify the specific role of CSM and/residues in CYP structure-function relationships. This information would be very useful for the design of CYP biocatalysts in order to improve activity and stability for industrial and medical purposes. A better understanding of the structure-function relationships in drug-metabolizing CYP enzymes will enable us to accurately predict drug metabolism and drug interaction.

## Figures and Tables

**Table 1:** Analysis of CYP mutants, locations, and functional characterizations.

CYP1A2	CSM	Region	Function	Reference
E163K, K170Q	6–7, 7	D helix	Enhanced activity Substrate selectivity	25–28
V193M	8–9	E helix		
E225N	10	F helix		
Q258H	11	G helix		
G437D	18	K′-L loop		
CYP2A6				
S183C	7–8	E helix	Enhanced activity Altered substrate specificity Novel activity	29–32
L206Q, F209I	8–9	F helix		
S224P	9	F-G loop		
L240C	10–11	G helix		
Y287H	11–12	I helix		
N297Q, I300V, T305S	12	I helix		
CYP2Bs				
V183L	7–8	E helix	Enhanced activity Altered substrate specificity Increased protein stability Enhanced tolerance to organic solvents	33–37
F202A, L209A	8–9	F helix		
K236I	10–11	G helix		
D257N	10–11	G-H loop		
L264F	11	H helix		
L295H	12	I helix		
S334P, P334S	13–14	J-J′ loop		
CYP3A4				
L216W	8	F helix	Enhanced activity Altered substrate specificity	38
F228I	10	F-G loop		
T433S	17	L-helix		

The location of the region is based on CYP X-ray structures and/or models of individual enzyme, except for CYP2Bs, in which CYP2B4 was used to identify regions. The CSM number is based on the CSM analysis of CYP2 family performed earlier (22). The information on functional characterizations of the mutants are based on earlier studies (last column) using rational and random mutagenesis approaches. Note that the regions for the same/similar residue numbers in different families (e.g. 1 vs. 2) vary more than the regions for the same/similar residue numbers within the same families or between different subfamilies (e.g. 2A vs. 2B). Represents between the two CSM or regions; e.g. 8–9 means between CSM 8 and 9.

**Table 2:** Identification and analysis of PCPMer motifs in CYP1 (19), CYP2 (175), and CYP3 (26) families in the E-H regions.

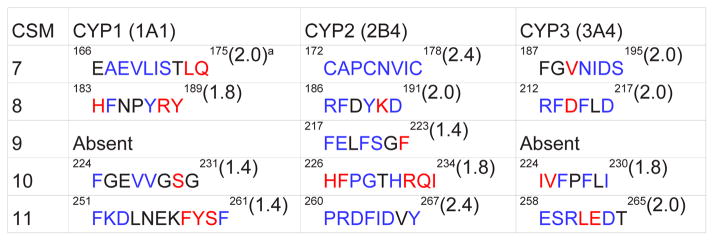

The number of CYP sequences analyzed for each family is shown in parenthesis

The CYP sequences used from various species are presented earlier (22)

The colors of the residues in the motifs represent the rank order of sequence conservation as a function of relative entropy; 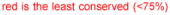, black is intermediate (75–90%), and 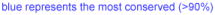

a The rank is shown in parenthesis

“Absent” in CYP1 and CYP2 represents lack of CSM 9 based on PCPMer motifs analysis.

Residues and their numbers in the motifs for each CYP family are based on the specific CYP indicated in the first row

**Table 3:** Identification and analysis of PCPMer motifs of CYP2A (12), CYP2B (10), CYP2C (33), and CYP2D (25) enzymes in the E-H region.

CSM	CYP2A (2A6)	CYP2B (2B4)	CYP2C (2C8)	CYP2D (2D6)
7	Absent	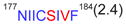	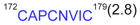	^180^KAVSN^184^
8	^193^YKDKEFLS^200^	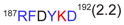	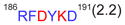	^192^GRRFEYDDP^200^
9	^217^GQLYEMFSSVM^227^	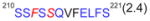	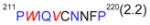	^225^NAVPVLLHIPALAGK^239^
10	Absent	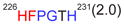	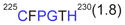	Absent
11	^260^DPNSP^264^	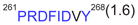	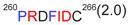	Absent

The number of CYP sequences analyzed for each family is shown in parenthesis

The CYP sequences used from various species are presented earlier (22)

The colors of the residues in the motifs represent the rank order of sequence conservation as a function of relative entropy; 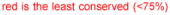, black is intermediate (75–90%), and 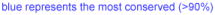

a The rank is shown in parenthesis. The rank of CYP2A and CYP2D are ≤1.2

“Absent” in CYP2A and CYP2D represents lack of their respective CSM based on PCPMer motifs analysis

Residues and their numbers in the motifs for each CYP2 subfamilies are based on the specific CYP indicated in the first row

**Table 4:** Comparison and analysis of CSM 7, 8, 10, and 11 sequences in important drug metabolizing human CYP enzymes.

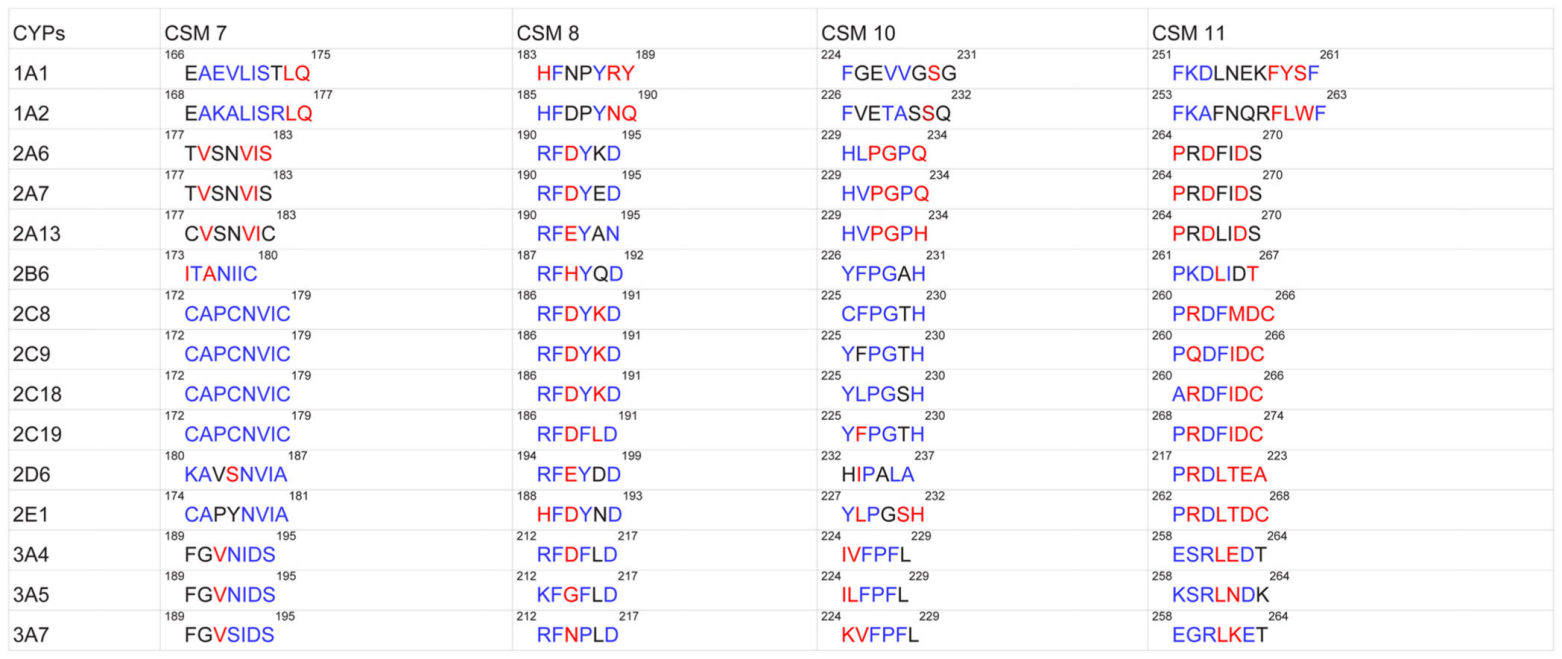

The CSM in all the CYPs is based on PCPMer motifs identified in the respective CYP1, CYP2, and CYP3 families (Table 2)

The colors of the residues in the motifs represent the rank order of sequence conservation as a function of relative entropy; 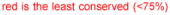, black is intermediate (75–90%), and 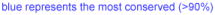

The order of residues conservation was determined manually using the known CYP sequences from various species in the respective families; 1A, 2A, 2B, 2C, 2D, 2E, and 3A

CSM 9 is not shown because it is not found in CYP1 and CYP3 families and is least conserved in CYP2 family
